# Effect of obstructive sleep apnea on prognosis in patients with acute coronary syndromes with varying numbers of standard modifiable risk factors: insight from the OSA-ACS study

**DOI:** 10.1007/s11239-023-02830-w

**Published:** 2023-05-27

**Authors:** Bin Wang, Yuekun Zhang, Wen Hao, Jingyao Fan, Yan Yan, Wei Gong, Wen Zheng, Bin Que, Hui Ai, Xiao Wang, Shaoping Nie

**Affiliations:** 1 Center for Coronary Artery Disease, Division of Cardiology, Beijing Anzhen Hospital, Capital Medical University, Beijing, China; 2 National Clinical Research Center for Cardiovascular Diseases, Beijing, China; 3 Emergency & Critical Care Center, Beijing Anzhen Hospital, Capital Medical University, Beijing, China; 4 Center for Coronary Artery Disease, Division of Cardiology, Beijing Anzhen Hospital, Capital Medical University, No. 2 Anzhen Road, Chaoyang District, Beijing, 100029 China

**Keywords:** Obstructive sleep apnea, Standard modifiable risk factor, Acute coronary syndrome, Prognostic outcomes

## Abstract

**Background:**

Standard modifiable risk factors (SMuRFs) increase the risk of cardiovascular events in patients with acute coronary syndrome (ACS) and are also strongly associated with obstructive sleep apnea (OSA) in a bidirectional relationship. However, the association of OSA with recurrent cardiovascular events in ACS patients based on the number of SMuRFs remains unclear. Hence, we aimed to elucidate the prognostic implication of OSA in ACS patients stratified by the number of SMuRFs.

**Methods:**

This was a post hoc analysis of the OSA-ACS study (NCT03362385), including 1927 patients admitted for ACS and undergoing portable sleep monitoring. OSA was defined as an apnea hypopnea index ≥ 15 events/h. The primary endpoint was major adverse cardiovascular and cerebrovascular event (MACCE) including cardiovascular death, myocardial infarction, stroke, hospitalization for unstable angina or heart failure, and ischemia-driven revascularization. Cox proportional hazards model and Kaplan-Meier analysis were used to investigated the relationship between OSA and subsequent cardiovascular events after patients were stratified by the number of SMuRFs.

**Results:**

Among 1927 patients enrolled, 130 (6.7%) had no SMuRF, 1264 (65.6%) exhibited 1–2 SMuRFs and 533 (27.7%) presented 3–4 SMuRFs. With the increase of the number of SMuRFs, the proportion of OSA in ACS patients tended to increase (47.7% vs. 51.5% vs. 56.6%), but there was no significant difference between them (P = 0.08). After the stratification of ACS patients via SMuRF numbers and adjustment for confounding factors, fully adjusted Cox regression indicated that OSA increased the risk of MACCE (adjusted HR, 1.65; 95%CI, 1.06–2.57; P = 0.026) and ischemia-driven revascularization (adjusted HR, 2.18; 95%CI, 1.03–4.65; P = 0.042) in ACS patients with 3–4 SMuRFs.

**Conclusions:**

In hospitalized ACS patients, OSA is associated with an increased risk of MACCE and ischemia-driven revascularization among patients with 3–4 SMuRFs. Therefore, screening for OSA should be emphasized in ACS patients with 3–4 SMuRFs, and intervention trials should be prioritized in these high-risk patients.

**Supplementary Information:**

The online version contains supplementary material available at 10.1007/s11239-023-02830-w.

## Introduction

Standard modifiable risk factors (SMuRFs), including diabetes, hypertension, hyperlipidemia, and smoking, are routinely targeted strategies for prevention and treatment of cardiovascular disease and are associated with higher risk of cardiovascular events in patients with acute coronary syndrome (ACS) [1–4]. Obstructive sleep apnea (OSA), which accounts for 40–60% of patients with cardiovascular disease, is a potential modifiable risk factor for cardiovascular disease characterized by intermittent hypoxia, activation of sympathetic nervous system, and sleep fragmentation [5, 6]. Accumulating studies have elucidated that OSA is an independent risk factor for long-term prognosis of cardiovascular disease [7, 8]. Additionally, a growing body of evidence suggests a bidirectional relationship between OSA and SMuRFs. OSA is an independent risk factor for hypertension [9], diabetes [10], and hyperlipidemia [11], while patients with these factors are also a susceptible population for OSA [12]. However, the prevalence and prognostic implication of OSA in ACS patients with different amounts of SMuRFs remain unclear. Therefore, we aimed to elucidate the prognostic implication of OSA in ACS patients stratified by the number of SMuRFs.

## Methods

### Study design and population

This is an ancillary study of the OSA-ACS project (NCT03362385), which is a single-center, prospective, cohort study aimed at investigating the association between OSA and cardio-cerebrovascular events of ACS patients [13–15]. In brief, patients included in the project were hospitalized for ACS in Beijing Anzhen Hospital, Capital Medical University from January 2015 and December 2019, whose age was in a range of 18 to 85 years old. Patients with cardiogenic shock, cardiac arrest, or malignant tumor were excluded. A sleep study failing or lasting for less than 180 min was also considered as an exclusion criterion. Additionally, patients were also excluded who are lost to follow-up, with central sleep apnea, or with management of regular continuous positive airway pressure. The study followed the STROBE (Strengthening the Reporting of Observational Studies in Epidemiology) guidelines [16], complied with the Declaration of Helsinki and was approved by the local committee (2,013,025). Meanwhile, all patients signed informed consent.

### Definition of SMuRFs

Standard modifiable risk factors include current smoking status, hypertension, hyperlipidemia, and diabetes [17]. Hypertension was defined as self-reported hypertension or using antihypertensive medication before admission; definition of hyperlipidemia referred to self-reported hyperlipidemia, using lipid-lowering medications before admission, an LDL-C concentration of 3.37 mmol/L or higher, or a total cholesterol concentration of 5.18 mmol/L; diabetes was defined as having a previous diagnosis of diabetes or previous glucose lowering pharmacotherapy. As both fasting glucose and acute phase blood pressure are influenced by neurohormonal response to admission status, these were not incorporated in the definitions.

### Procedure and management

Portable cardiorespiratory polygraphy (Apnea-Link, ResMed, Australia) was applied to record the situation of arterial oxygen saturation, nasal airflow, snoring episodes, and thoracoabdominal movements in order to study nocturnal sleep-in stabilized patients. It was considered as a valid test when the polygraph recording was satisfactory and lasted at least 3 h. Airflow absence lasting ≥ 10 s was identified as apnea that was classified into obstructive apnea with thoracoabdominal movement and central apnea without thoracoabdominal movement. Hypopnea was defined as > 30% reduction in airflow for ≥ 10 s accompanied by a > 4% decrease in arterial oxygen saturation. Apnea-hypopnea index (AHI) refers to the cumulative number of apneas and hypopneas per hour during the total recording time. All parameters of sleep study were judged by two independent sleep technologists, while in cases of contradiction, further judgement was performed by a senior sleep medicine consultant. Recruited patients were stratified into OSA group (AHI ≥ 15 events per hour) and non-OSA group (AHI < 15 events per hour). All patients received guideline-directed medical therapy and if necessary, percutaneous coronary intervention or coronary artery bypass grafting was performed. Unless contraindicated, patients discharged from the hospital were administered dual antiplatelet therapy (aspirin plus clopidogrel or ticagrelor) for at least 1 year. Referral to sleep center was offered to patients with moderate to severe sleep apnea (AHI ≥ 15), especially those with excessive daytime sleepiness, in order to achieve further evaluation and intervention.

### Follow-up and outcomes

After discharge, follow-up was performed at the clinic or via telephone at 1 month, 3 months, 6 months, 1 year, and then every 6 months. Major adverse cardiovascular and cerebrovascular events (MACCE) were the primary endpoints that included cardiovascular death, ischemia-driven revascularization, myocardial infarction, hospitalization for unstable angina or heart failure, and stroke. Secondary endpoints included components of MACCE, all repeat revascularization, a composite of cardiovascular death, myocardial infarction, or ischemic stroke, and a composite consisting of cardiac events which was the components of the primary end point after excluding stroke.

### Statistical analysis

Quantitative data with normal distribution were described by mean ± standard deviation and compared via analysis of variance while non-normal distributed data were described by median (interquartile range) and the difference was analyzed via Kruskal-Walls test. The description of qualitative data was performed by frequency and percentage and the difference was discriminated by chi-square tests. Cox proportional hazards model was utilized to analyze the relationship between OSA and endpoint events after stratification of patients into subgroups according to the number of SMuRFs. Model covariates were determined based on clinical relevance or univariate relationships between variables and outcomes. Finally, we applied 3 models in the study: (1) unadjusted; (2) partially adjusted for age and sex; (3) fully adjusted for age (per 10-year increase), sex, body mass index, prior stroke, prior myocardial infarction, left ventricular ejection fraction (LVEF) less than 40%, systolic blood pressure (per 10 mm Hg increase), and plasma creatinine (per 10 µmol/L increase). Statistical analysis was performed via SPSS (version 26.0 IBM SPSS Inc, Armonk, NY) and a two-side P value less than 0.05 was the standard of significant difference.

## Results

### Baseline characteristics

**Fig. 1:**
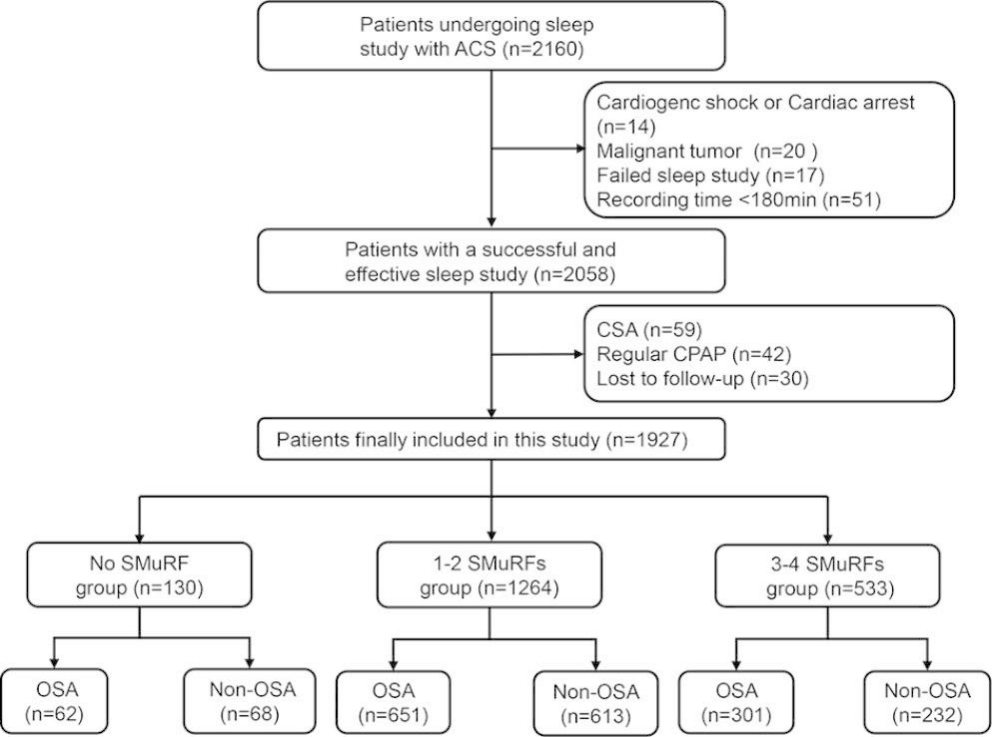
Study Flowchart ACS, acute coronary syndrome; CPAP, continuous positive airway pressure; CSA, central sleep apnea; OSA, obstructive sleep apnea; SMuRFs, standard modifiable risk factors

The project included 2016 ACS patients undergoing sleep study, of whom 2058 patients completed a sleep study and acquired valid results. After further screening based on exclusion criteria, we eventually enrolled 1927 patients for the final analysis (Fig. 1), among whom 130(6.7%) patients had no SMuRF, 1264(65.6%) patients had 1–2 SMuRFs and 533(27.7%) patients had 3–4 SMuRFs. The clinical characteristics of patients stratified by the number of SMuRFs was presented in Table 1. Patients with more SMuRFs were younger (*P* = 0.004), meanwhile, while with the accumulation of SMuRFs, there was an increase in body mass index (*P* < 0.001), waist to hip ratio (*P* < 0.001) and Hs-CRP (*P* = 0.03). Additionally, as the number of SMuRFs increased, patients with diabetes, hypertension, hyperlipidemia, and prior stroke accounted for a greater proportion and there were more current smokers and alcohol drinkers.

**Table 1:** Baseline Clinical Characteristics by number of cardiovascular risk factors

Variables	All (N = 1927)	No SMuRF (N = 130)	1–2 SMuRFs (N = 1264)	3–4 SMuRFs (N = 533)	P value
Demographics					
Age, mean ± SD, years	56.4 ± 10.5	57.2 ± 10.8	56.9 ± 10.3	55.0 ± 10.7	0.004
Male	1629 (84.5)	109 (83.8)	1061 (83.9)	459 (86.1)	0.49
BMI, mean ± SD, kg/m^2^	27.1 ± 3.6	26.2 ± 2.8	26.9 ± 3.6	27.8 ± 3.7	p < 0.001
Waist-to-hip ratio, median (IQR)	0.98 (0.95–1.02)	0.97 (0.94-1.00)	0.98 (0.94–1.01)	0.99 (0.96–1.03)	p < 0.001
Neck circumference, median (IQR), cm	41 (38–43)	40 (37–42)	40 (38–43)	41 (39–44)	p < 0.001
Systolic BP, median (IQR), mmHg	126 (117–138)	123 (113–130)	125 (116–138)	130 (120–140)	p < 0.001
Diastolic BP, median (IQR), mmHg	76 (70–84)	73 (69–80)	76 (70–84)	78 (70–86)	0.002
**Medical History**					
Diabetes	609 (31.6)	0 (0)	246 (19.5)	363 (68.1)	p < 0.001
Hypertension	1247 (64.7)	0 (0)	752 (59.5)	495 (92.9)	p < 0.001
Hyperlipidemia	637 (33.1)	0 (0)	301 (23.8)	336 (63.0)	p < 0.001
Family history of premature CAD	104 (5.4)	5 (3.8)	62 (4.9)	37 (6.9)	0.16
Prior stroke	207 (10.7)	5 (3.8)	124 (9.8)	78 (14.6)	p < 0.001
Prior myocardial infarction	316 (16.4)	20 (15.4)	211 (16.7)	85 (15.9)	0.88
Prior PCI	399 (20.7)	22 (16.9)	264 (20.9)	113 (21.2)	0.54
Prior CABG	29 (1.5)	1 (0.8)	18 (1.4)	10 (1.9)	0.60
Smoking					p < 0.001
No	654 (33.9)	84 (64.6)	475 (37.6)	95 (17.8)	
Current	913 (47.4)	0 (0)	519 (41.1)	394 (73.9)	
Previous	360 (18.7)	46 (35.4)	270 (21.4)	44 (8.3)	
Drinking					p < 0.001
No	1181 (61.3)	104 (80.0)	786 (62.2)	291 (54.6)	
Current	637 (33.1)	18 (13.8)	398 (31.5)	221 (41.5)	
Previous	109 (5.7)	8 (6.2)	80 (6.3)	21 (3.9)	
**Baseline Tests**					
eGFR, median (IQR), mL/min/1.73 m^2^	104.9 (89.4-121.2)	105.2 (91.4-122.6)	105.8 (89.3-120.3)	105.3 (88.6-122.4)	0.51
Hs-CRP, median (IQR), mg/L	2.0 (0.8–6.1)	1.2 (0.7–4.4)	1.9 (0.8–6.2)	2.3 (0.9–6.1)	0.03
LVEF, median (IQR), %	61 (56–65)	61 (55–66)	62 (56–65)	61 (56–65)	0.88

Data are presented as mean ± SD, median (IQR), or n (%). BMI, body mass index; BP, blood pressure; CABG, coronary artery bypass grafting; CAD, coronary artery disease; eGFR, glomerular filtration rate; Hs-CRP, high-sensitivity C-reactive protein; IQR, interquartile range; LVEF, left ventricular ejection fraction; OSA, obstructive sleep apnea; PCI, percutaneous coronary intervention; SD, standard deviation; SMuRF, standard modifiable risk factor

As depicted in Table 2 and Supplementary material, Figure S1, the proportion of patients without SMuRF combined with OSA was 47.7% and the proportion of patients with 1–2 SMuRF combined with OSA was 51.5%. Besides, the proportion of patients with 3–4 SMuRF combined with OSA was 56.6%. This presented a trend that the proportion of patients combined OSA increased with the increase of the number of SMURFs, but there was no statistical difference (*P* = 0.08). The median AHI was 14.5 (6.6–23.1), 15.6 (7.0-28.8) and 18.0 (8.5–36.2) events per hour among patients with no SMuRF, 1–2 SMuRFs, and 3–4 SMuRFs, respectively (*P* = 0.001). Compared with patients without SMuRFs, patients with more SMuRFs exhibited higher oxygen desaturation index (ODI) (*P* = 0.004) and longer time of arterial oxygen saturation less than 90% (*P* < 0.001), accompanied by a reduction of nadir arterial oxygen saturation(*P* < 0.001). Furthermore, patients with more SMuRFs were more probable to be administered the treatment of ACEIs/ARB (42.3% versus 60.0% versus 71.7, *P* < 0.001). Participants were also stratified by OSA to further describe their characteristics, as shown in Supplementary material, table S1 and table S2.

**Table 2:** Clinical Presentations and Management by number of cardiovascular risk factors

Variables	All (N = 1927)	No SMuRF (N = 130)	1–2 SMuRFs (N = 1264)	3–4 SMuRFs (N = 533)	P value
**Diagnosis**					0.93
STEMI	430 (22.3)	28 (21.5)	290 (22.9)	112 (21.0)	
NSTEMI	365 (18.9)	24 (18.5)	238 (18.8)	103 (19.3)	
Unstable angina	1132 (58.7)	78 (60.0)	736 (58.2)	318 (59.7)	
**Procedures**					
Coronary angiography	1877 (97.4)	129 (99.2)	1227 (97.1)	521 (97.7)	0.28
Revascularization	1335 (69.3)	86 (66.2)	868 (68.7)	381 (71.5)	0.36
PCI	1209 (62.7)	79 (60.8)	787 (62.3)	343 (64.4)	0.63
DES use	1051 (86.9)	67 (51.5)	688 (54.4)	296 (55.5)	0.71
Baseline TIMI 0 or 1	422 (34.9)	20 (25.3)	282 (35.8)	120 (35.0)	0.17
Final TIMI 3	1189 (98.3)	78 (98.7)	776 (98.6)	335 (97.7)	0.51
CABG	130 (6.7)	8 (6.2)	84 (6.6)	38 (7.1)	0.89
**Sleep Study**					
OSA	1014 (52.6)	62 (47.7)	651 (51.5)	301 (56.6)	0.08
AHI, median (IQR), events·h^− 1^	16.0 (8.0–30.0)	14.5 (6.6–23.1)	15.6 (7.9–28.8)	18.0 (8.5–36.2)	0.001
ODI, median (IQR), events·h^− 1^	16.2 (8.8–28.6)	14.1 (8.0-24.7)	16.2 (8.7–27.4)	17.2 (9.3–33.2)	0.004
Nadir SaO_2_, median (IQR), %	85 (81–88)	87 (83–89)	85 (81–88)	84 (79–88)	p < 0.001
Mean SaO_2_, median (IQR), %	94 (93–95)	94 (93–95)	94 (93–95)	94 (92–95)	0.06
Time with SaO_2_ < 90%, median (IQR), %	2.3 (0.4–10.0)	1.0 (0.2–5.3)	2.0 (0.3–9.3)	3.1 (0.6–12.0)	p < 0.001
Epworth Sleepiness Scale, median (IQR)	7.0 (4.0–11.0)	7.0 (2.0-9.3)	7.0 (4.0–11.0)	9.0 (5.0–12.0)	0.03
**Medications on Discharge**					
Aspirin	1877 (97.4)	124 (95.4)	1239 (98)	514 (96.4)	0.05
P2Y_12_ inhibitors	1768 (91.7)	118 (90.8)	1162 (91.9)	488 (91.6)	0.88
β-Blockers	1488 (77.2)	99 (76.2)	966 (76.4)	423 (79.4)	0.38
ACEIs/ARBs	1195 (62.0)	55 (42.3)	758 (60.0)	382 (71.7)	p < 0.001
Statins	1897 (98.4)	127 (97.7)	1251 (99.0)	519 (97.4)	0.034

Data are presented as mean ± SD, median (IQR), n (%), or n/N (%). ACEI, angiotensin-converting enzymes inhibitor; AHI, apnea-hypopnea index; ARB, angiotensin receptor blocker; CABG, coronary artery bypass grafting; DES, drug eluting stent; Hs-CRP, high-sensitivity C-reactive protein; IQR, interquartile range; NSTEMI, non-ST-segment elevation myocardial infarction; ODI, oxygen desaturation index; OSA, obstructive sleep apnea; PCI, percutaneous coronary intervention; SaO_2_, arterial oxygen saturation; SD, standard deviation; SMuRF, standard modifiable risk factor; STEMI, ST-segment-elevation myocardial infarction; TIMI, thrombolysis in myocardial infarction

### Effect of different number of risk factors on prognosis of ACS

The median of follow-up for the study was 34.97 (18.67–43.23) months. Kaplan-Meier analysis elucidated no difference in the cumulative incidence of MACCE for patients with different numbers of SMuRFs (Log-rank, *P* = 0.58; ***Supplementary material, Figure S2*)**. Notably, during the late follow-up period, Kaplan-Meier analysis presented a growing trend towards cumulative incidence of MACCE with increasing number of SMuRFs. Data about crude number of all events in patients stratified by the number of SMuRFs were shown in ***Supplementary material, Table S3***.

### Outcomes of OSA versus non‑OSA patients stratified by number of SMuRFs

**Fig. 2:**
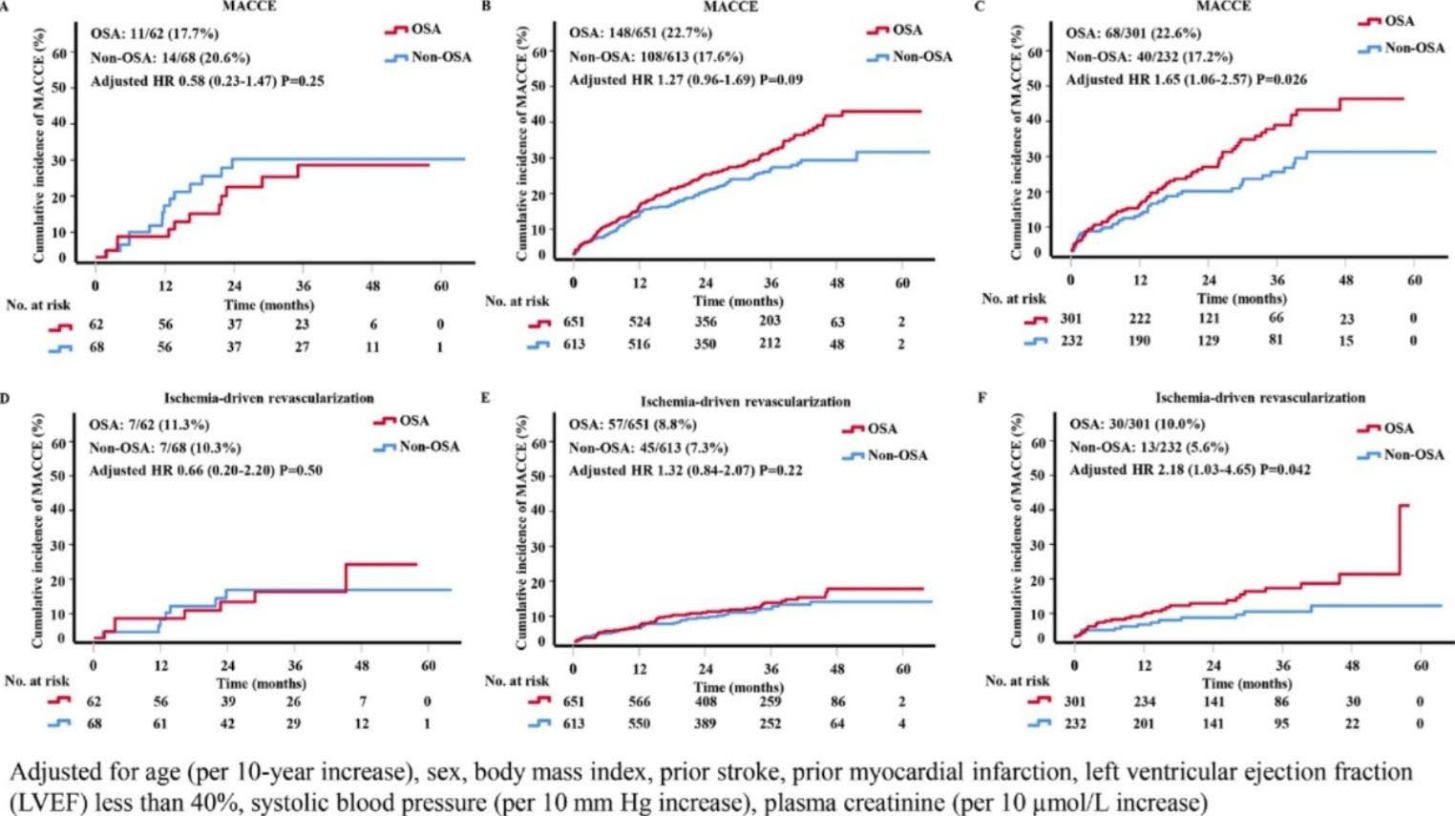
Cumulative Incidence of MACCE and Ischemia-driven Revascularization by SMuRFs and OSA Categories Kaplan-Meier estimates and fully-adjusted HR for MACCE and Ischemia-driven Revascularization in patients with No SMuRF (A, D), 1-2SMuRFs (B, E), or 3–4 SMuRFs (C, F). Adjusted for age (per 10-year increase), sex, body mass index, prior stroke, prior myocardial infarction, left ventricular ejection fraction (LVEF) less than 40%, systolic blood pressure (per 10 mm Hg increase), plasma creatinine (per 10 µmol/L increase). MACCE, major adverse cardiovascular and cerebrovascular events; OSA, obstructive sleep apnea; SMuRFs, standard modifiable risk factors;

The correlation between OSA and risk of cardiovascular events was analyzed by cox regression stratified by number of SMuRFs. As depicted in Tables 3***and*** Fig. 2, OSA in the 3–4 SMuRFs group had a significant influence on the incidence of MACCE (adjusted *HR*, 1.65; 95%*CI*, 1.06–2.57; *P* = 0.026) and ischemia-driven revascularization (adjusted *HR*, 2.18; 95%*CI*, 1.03–4.65; *P* = 0.042), after adjusted for age (per 10-year increase), sex, body mass index, prior stroke, prior myocardial infarction, left ventricular ejection fraction (LVEF) less than 40%, systolic blood pressure (per 10 mm Hg increase), and plasma creatinine (per 10 µmol/L increase). Data about crude number of all events in OSA or non-OSA patients overall or stratified by the number of SMuRFs were shown in Supplementary material, Table S4.

**Table 3:** Cox Regression Analyses Evaluating the Association Between OSA and Risk of Cardiovascular Events by number of cardiovascular risk factors

Outcomes	Unadjusted		Partially Adjusted*		Fully Adjusted†	
	HR (95% CI)	P value	HR (95% CI)	P value	HR (95% CI)	P value
MACCE						
No SMuRF	0.81 (0.37–1.79)	0.61	0.77 (0.35–1.72)	0.53	0.58 (0.23–1.47)	0.25
1–2 SMuRFs	1.33 (1.04–1.71)	0.02	1.32 (1.03–1.69)	0.03	1.27 (0.96–1.69)	0.09
3–4 SMuRFs	1.54 (1.04–2.27)	0.03	1.58 (1.07–2.34)	0.02	1.65 (1.06–2.57)	0.026
Cardiovascular death						
No SMuRF	-	-	-	-	-	-
1–2 SMuRFs	2.16 (0.89–5.26)	0.09	2.00 (0.82–4.88)	0.13	1.64 (0.58–4.62)	0.35
3–4 SMuRFs	0.49 (0.12–2.04)	0.33	0.42 (0.10–1.81)	0.25	0.38 (0.08–1.88)	0.38
Myocardial infarction						
No SMuRF	1.33 (0.08–21.4)	0.84	1.12 (0.07–18.2)	0.94	-	-
1–2 SMuRFs	1.53 (0.77–3.06)	0.23	1.49 (0.74–2.98)	0.26	1.44 (0.63–3.27)	0.39
3–4 SMuRFs	2.61 (0.83–8.20)	0.10	2.69 (0.85–8.48)	0.09	1.52 (0.44–5.29)	0.51
Stroke						
No SMuRF	0.55 (0.05–6.07)	0.63	0.62 (0.06–7.04)	0.70	0.72 (0.01–43.5)	0.88
1–2 SMuRFs	1.27 (0.54–3.01)	0.59	1.22 (0.51–2.90)	0.66	1.27 (0.47–3.47)	0.64
3–4 SMuRFs	1.48 (0.58–3.77)	0.41	1.51(0.59–3.82)	0.39	1.83 (0.62–5.38)	0.28
Hospitalization for unstable angina						
No SMuRF	0.87 (0.36–2.09)	0.75	0.80 (0.33–1.96)	0.64	0.64 (0.23–1.77)	0.39
1–2 SMuRFs	1.22 (0.91–1.64)	0.18	1.23 (0.92–1.65)	0.17	1.21 (0.87–1.68)	0.27
3–4 SMuRFs	1.53 (0.94–2.48)	0.09	1.58 (0.97–2.57)	0.07	1.75 (1.01–3.04)	0.046
Hospitalization for heart failure						
No SMuRF	-	-	-	-	-	-
1–2 SMuRFs	0.67 (0.23–1.94)	0.46	0.62 (0.21–1.79)	0.37	0.33 (0.08–1.33)	0.33
3–4 SMuRFs	1.76 (0.32–9.63)	0.52	1.62 (0.29–8.94)	0.58	1.93 (0.30–12.3)	0.49
Ischemia-driven revascularization						
No SMuRF	1.10 (0.38–3.13)	0.86	1.07 (0.37–3.10)	0.90	0.66 (0.20–2.20)	0.50
1–2 SMuRFs	1.21 (0.82–1.79)	0.34	1.22 (0.83–1.81)	0.32	1.32 (0.84–2.07)	0.22
3–4 SMuRFs	2.01 (1.05–3.86)	0.036	2.00 (1.04–3.84)	0.038	2.18 (1.03–4.65)	0.042
Composite for cardiovascular death, myocardial infarction, or ischemic stroke						
No SMuRF	0.58 (0.11–3.18)	0.53	0.59 (0.11–3.20)	0.54	0.64 (0.07–5.92)	0.70
1–2 SMuRFs	1.76 (1.09–2.84)	0.02	1.68 (1.04–2.71)	0.033	1.74 (1.00-3.06)	0.052
3–4 SMuRFs	1.44 (0.77–2.69)	0.25	1.49 (0.79–2.78)	0.22	1.21 (0.60–2.43)	0.60
Composite for cardiac events§						
No SMuRF	0.88 (0.38–2.03)	0.76	0.81 (0.35–1.88)	0.62	0.56 (0.21–1.50)	0.25
1–2 SMuRFs	1.33 (1.03–1.71)	0.03	1.31 (1.01–1.70)	0.04	1.26 (0.94–1.69)	0.12
3–4 SMuRFs	1.46 (0.96–2.23)	0.08	1.50 (0.98–2.29)	0.06	1.51 (0.94–2.43)	0.09
All repeat revascularization						
No SMuRF	0.99 (0.42–2.33)	0.98	0.93 (0.39–2.20)	0.86	0.71 (0.26–1.88)	0.49
1–2 SMuRFs	1.28 (0.92–1.79)	0.15	1.28 (0.91–1.79)	0.16	1.36 (0.93–1.99)	0.12
3–4 SMuRFs	1.20 (0.73–1.96)	0.47	1.17 (0.72–1.92)	0.53	1.34 (0.76–2.37)	0.32

*Adjusted for age and sex. †Adjusted for age (per 10-year increase), sex, body mass index, prior stroke, prior myocardial infarction, left ventricular ejection fraction (LVEF) less than 40%, systolic blood pressure (per 10 mm Hg increase), plasma creatinine (per 10 µmol/L increase). §Include cardiovascular death, myocardial infarction, ischemia-driven revascularization, or hospitalization for unstable angina or heart failure. CI, confidence interval; HR, hazard ratio; MACCE, major adverse cardiovascular and cerebrovascular event; OSA, obstructive sleep apnea; SMuRF, standard modifiable risk factor

## Discussion

In the study, we found that with the accumulation of SMuRFs, the symptom of OSA presented a more frequent incidence of apnea and hypopnea and a reduced oxygen saturation with a longer duration, which elucidated that patient with more SMuRFs had a worse situation of OSA. In hospitalized ACS patients, OSA is associated with an increased risk of MACCE and ischemia-driven revascularization among patients with 3–4 SMuRFs.

### Hypoxia evoked by OSA in ACS patients with SMuRFs

Our study founded that the proportion of OSA in ACS patients exhibited an increased trend with the accumulation of SMuRFs (47.7% versus 51.5% versus 56.6%), but there was no significant difference (P = 0.08). Nonetheless, the higher AHI and ODI, the longer time of arterial oxygen saturation less than 90%, and the reduction of nadir arterial oxygen saturation elucidated the potential worsening hypoxia evoked by OSA in ACS patients with more SMuRFs. Hypoxia during sleep is one of the distinguished characteristics of OSA pathophysiological process [18]. The repetitive process of desaturation and reoxygenation named intermittent hypoxia in patients with OSA activated the hypoxia-sensitive transcription factor-1 and NF-kB [19]. After the activation, adhesion factors are up-regulated, eliciting the recruitment and migration of macrophage, facilitating the inflammation process [20]. Eventually, hypoxia caused by OSA contributes to coronary atherosclerosis and vascular events [21, 22]. Therefore, given that the exacerbation of hypoxia caused by OSA with the accumulation of SMuRFs, we hypothesized that OSA might insult an increased incidence of cardio- cerebrovascular events in ACS patients with a greater number of SMuRFs.

### OSA as a synergistic risk factor in ACS patients with 3–4 SMuRFs

It is acknowledged that the accumulation of SMuRFs means an increased burden that is implicated with a greater risk of cardiovascular events [23]. In contrast, some previous research held a different view that the absence of SMuRFs contributed to a higher mortality in patients with acute coronary syndrome [24–26]. Figtree et al. argued that fewer application of drugs improving prognosis, including ACEI/ARB, β-blocker, and statin, accounted for the controversy, after adjusting for which their results elucidated that patient with more SMuRFs conferred an increased risk of mortality in patients with segment elevated myocardial infarction [27]. Patients without SMuRFs had lower odds of all cause and cardiac mortality and major adverse cardiovascular events after propensity score matching [17].

Although there exists no study on the association between OSA and SMuRFs, previous literatures have probed the impact of OSA on clinical outcomes in patients with heart disease complicated by diabetes, hypertension, or hyperlipidemia [28]. The severity of OSA is an independent risk factor for insulin resistance via the activation of the hypothalamus-adrenal axis, increasing the cardiovascular disease risk [29]. Previous studies have also demonstrated that diabetes patients with OSA had a higher proportion of microvascular complications and increased the risk of MACE, unstable angina hospitalization, and all-cause mortality [30–32]. Therefore, OSA is potentially a synergistic risk factor for diabetes [33]. Hypoxia induced by OSA promotes systemic inflammation and oxidative stress, which results in the upregulation of endothelin-1 and decrease of nitric oxide in endothelial cells, insulting increased arterial peripheral resistance and elevation of blood pressure; besides, OSA also affects elevation of blood pressure via activating sympathetic nerve. Eventually, OSA accelerates the adverse cardiovascular remodeling in patients with resistant hypertension [34]. OSA also contributes to dyslipidemia via upregulating involved enzymes to induce the increase of liver lipid synthesis [35].

In our study, OSA was in correlation with an increased incidence of MACCE and ischemia-driven revascularization events in ACS patients with 3–4 SMuRFs after adjusting for confounding factors and hence we think OSA serves as a synergistic risk factor in ACS patients with 3–4 SMuRFs and it is necessary to screen for OSA in ACS patients, especially in whom have more SMuRFs.

### Intervention of OSA in ACS patients with SMuRFs

Although OSA is an independent risk factor for cardiovascular events in ACS patients, the effect of continuous positive airway pressure (CPAP) on secondary cardiovascular protection is controversial. Previous literature elucidated that CPAP showed no reduction in the incidence of cardiovascular events [36], while Fuji Yoshi et al. showed that CPAP reduces the incidence of cardiovascular events and the accumulation of macrophage in culprit lesions [37]. The difference might be attributed to the phenotype of OSA, for instance, the phenotype of OSA in patients with coronary artery disease and diabetes is at high risk of cardiovascular events and benefits from the treatment of CPAP [38]. Given the synergistic effect of OSA in ACS patients with SMuRFs and the reduced influence of OSA intervention on cardiovascular risk factors, such as blood pressure [39] and blood glucose [40], we recommend that OSA in ACS patients with SMuRFs should receive the treatment of CPAP. The lack of relevant researches prompt further research to explore the benefits of CPAP for the ACS population with SMURFs.

### Limitations

There existed some limitations in this study. First, we conducted this study in ACS patients while OSA severity was potentially overestimated in any high-risk disease setting. Second, we assessed the OSA with the portable polygraphy which possibly underestimated the severity caused by overestimation of actual sleeping time. Third, this study is based on a prospective cohort study, which inevitably affects the results due to its own disadvantages. Finally, this study was an observational, single-center study recruiting patients from China, which made it necessary to further validate the conclusion in cohort studies and limited the extension to more ethic population.

## Conclusions

In hospitalized ACS patients, OSA is associated with an increased risk of MACCE and ischemia-driven revascularization among patients with 3–4 SMuRFs. Therefore, screening for OSA should be emphasized in ACS patients with 3–4 SMuRFs, and intervention trials should be prioritized in these high-risk patients.

## Electronic supplementary material

Below is the link to the electronic supplementary material.

Supplementary Material 1

## Data Availability

Data collected and analyzed in this study are included in this article or the supplementary material files.
