# Supplementary material for: Effect of obstructive sleep apnea on prognosis in patients with acute coronary syndromes with varying numbers of standard modifiable risk factors: insight from the OSA-ACS study
Source: J Thromb Thrombolysis. 2023 May 27;56(1):65–74. doi: 10.1007/s11239-023-02830-w (PMC10284994; doi:10.1007/s11239-023-02830-w)

Effect of obstructive sleep apnea on prognosis in patients with acute coronary syndromes with varying numbers of standard modifiable risk factors: Insight from the OSA-ACS study

Running Title: Comorbid OSA and SMuRFs with ACS Outcomes

Journal name：Journal of Thrombosis and Thrombolysis

Bin Wang, MD[a,b,#]; Yuekun Zhang, MD[a,b,#]; Wen Hao, MD[a,b]; Jingyao Fan, MD[c,b]; Yan Yan, MD[a,b]; Wei Gong, MD, PhD[a,b]; Wen Zheng, MD, PhD[a,b]; Bin Que, MD[a,b]; Hui Ai, MD[a,b], Xiao Wang, MD[a,b,*]; Shaoping Nie, MD, PhD[a,b,*]

[a]Center for Coronary Artery Disease, Division of Cardiology, Beijing Anzhen Hospital, Capital Medical University, Beijing, China
[b]National Clinical Research Center for Cardiovascular Diseases, Beijing, China
[c]Emergency & Critical Care Center, Beijing Anzhen Hospital, Capital Medical University, Beijing, China

[#]Bin Wang and YueKun Zhang contributed equally to this work.
*Xiao Wang and Shaoping Nie were co-senior authors

Corresponding author:

Shaoping Nie, MD, PhD
Center for Coronary Artery Disease, Division of Cardiology
Beijing Anzhen Hospital, Capital Medical University
No. 2 Anzhen Road, Chaoyang District
Beijing, China 100029
E-mail: spnie@ccmu.edu.cn

Xiao Wang, MD
Center for Coronary Artery Disease, Division of Cardiology
Beijing Anzhen Hospital, Capital Medical University.
No. 2 Anzhen Road, Chaoyang District
Beijing, China 100029
E-mail: spaceeye123@126.com

Table S1. Baseline Clinical Characteristics of OSA versus non-OSA groups overall and in patients with 3-4 SMuRFs.

	All (N=1927)			3-4 SMuRFs (N = 533)		
**Variables**	**OSA (n=1014)**	**Non-OSA (n=913)**	***P* value**	**OSA (n=301)**	**Non-OSA(n=232)**	***P* value**
**Demographics**						
Age, mean±SD, years	56.52±10.56	56.22±10.41	0.526	54.90±10.89	55.20±10.47	0.744
Male	886(87.4)	743(81.4)	<0.001	265(88.0)	194(83.6)	0.143
BMI, mean±SD, kg/m^2	28.05±3.55	25.99±3.40	<0.001	28.85±3.65	26.43±3.25	<0.001
Waist-to-hip ratio, median (IQR)	0.99 (0.96, 1.00)	0.97(0.94, 1.00)	<0.001	1.00(0.96, 1.03)	0.98(0.95, 1.01)	<0.001
Neck circumference, median (IQR), cm	41.5 (39.0, 44.0)	40.00(37.5, 42.0)	<0.001	42.5(41.0,45.0)	40.0(38.0, 42.0)	<0.001
Systolic BP, median (IQR), mmHg	126(117, 139)	126(117, 138)	0.345	130(120, 139.5)	130(120, 141)	0.693
Diastolic BP, median (IQR), mmHg	77(70, 86)	75(69, 83)	<0.001	79(70, 87)	75(70, 84)	0.006
**Medical History**						
Diabetes	319(31.5)	290(31.8)	0.886	201(66.8)	162(69.8)	0.454
Hypertension	691(68.1)	556(60.9)	<0.001	285(94.7)	210(90.5)	0.064
Hyperlipidemia	343(33.8)	294(32.2)	0.449	192(63.8)	144(62.1)	0.684
Family history of premature CAD	51(5.0)	53(5.8)	0.452	17(5.6)	20(8.6)	0.181
Prior stroke	121(11.9)	86(9.4)	0.075	52(17.3)	26(11.2)	0.049
Prior myocardial infarction	177(17.5)	139(15.2)	0.187	56(18.6)	29(12.5)	0.056
Prior PCI	234(23.1)	165(18.1)	0.007	71(23.6)	42(18.1)	0.125
Prior CABG	18(1.8)	11(1.2)	0.305	7(2.3)	3(1.3)	0.583
Smoking			0.360			0.729
No	333(32.8)	321(35.2)		55(18.3)	40(17.2)	

Current	496(48.9)	417(45.7)		219(72.8)	175(75.4)	
Previous	185(18.2)	175(19.2)		27(9.0)	17(7.3)	
Drinking			0.005			0.344
No	589(58.1)	592(64.8)		157(52.2)	134(57.8)	
Current	357(35.2)	280(30.7)		130(43.2)	91(39.2)	
Previous	68(6.7)	41(4.5)		14(4.7)	7(3.0)	
**Baseline Tests**						
eGFR, median (IQR), mL/min/1.73 m^2	103.10(87.55,118.90)	107.08(90.76,123.48)	0.003	104.48(88.54,118.97)	107.47(89.23,126.38)	0.162
Hs-CRP, median (IQR), mg/L	2.50(1.03, 7.35)	1.45(0.61, 4.44)	<0.001	2.89(1.19, 6.93)	1.70(0.70,4.09)	<0.001
LVEF, median (IQR), %	61.0(55.0, 65.0)	62.0(57.0, 66.0)	0.009	60.0(55.0, 65.0)	62.0(58.0, 65.0)	0.013

Data are presented as mean±SD, median (IQR), or n (%). BMI, body mass index; BP, blood pressure; CABG, coronary artery bypass grafting; CAD, coronary artery disease; eGFR, glomerular filtration rate; Hs-CRP, high-sensitivity C-reactive protein; IQR, interquartile range; LVEF, left ventricular ejection fraction; OSA, obstructive sleep apnea; PCI, percutaneous coronary intervention; SD, standard deviation.

Table S2. Clinical Presentations and Management of OSA versus non-OSA groups overall and in patients with 3-4 SMuRFs.

	All (N=1927)			3-4 SMuRFs (N = 533)		
**Variables**	**OSA (n=1014)**	**Non-OSA (n=913)**	***P* value**	**OSA (n=301)**	**Non-OSA (n=232)**	***P* value**
**Diagnosis**			0.021			0.461
STEMI	251(24.8)	179(19.6)		69(22.9)	43(18.5)	
NSTEMI	191(18.8)	174(19.1)		56(18.6)	47(20.3)	
Unstable angina	572(56.4)	560(61.3)		176(58.5)	142(61.2)	
**Procedures**						
Coronary angiography	990(97.6)	887(97.2)	0.507	295(98.0)	226(97.4)	0.647
Revascularization	725(71.5)	610(66.8)	0.026	216(71.8)	165(71.1)	0.871
PCI	667(65.8)	542(59.4)	0.004	196(65.1)	147(63.4)	0.675
DES use	581(57.3)	470(51.5)	0.010	167(55.5)	129(55.6)	0.978
Baseline TIMI 0 or 1	244(36.6)	178(32.8)	0.175	70(35.7)	50(34.0)	0.744
Final TIMI 3	654(98.1)	535(98.7)	0.373	190(96.9)	145(98.6)	0.502
CABG	59(5.8)	71(7.8)	0.087	20(6.6)	18(7.8)	0.620
**Sleep Study**						
AHI, median (IQR), events·h^{-1}	29.2(20.8, 42.1)	7.6(4.2, 10.8)	<0.001	33.4(22.1,46.7)	7.6(4.2, 10.7)	<0.001
ODI, median (IQR), events·h^{-1}	27.5(20.2, 39.7)	8.6(4.8, 11.9)	<0.001	30.9(21.7,44.5)	8.6(5.1, 11.4)	<0.001
Nadir SaO_2, median (IQR), %	83(77, 86)	87(84, 90)	<0.001	81(76,86)	87(84, 89)	<0.001
Mean SaO_2, median (IQR), %	93(92. 94)	94(93, 95)	<0.001	93(92,94)	94(93, 95)	<0.001
Time with SaO_2<90%, median (IQR), %	6.0(2.0, 16.0)	0.6(0.1, 3.0)	<0.001	8.0(2.4,21.0)	0.9(0.1, 3.0)	<0.001
Epworth Sleepiness Scale, median (IQR)	8(5, 12)	6(3, 10)	<0.001	9(6, 13)	7(3, 11)	0.014
**Medications on Discharge**						

Aspirin	987(97.3)	890(97.5)	0.843	290(96.3)	224(96.6)	0.899
$P2Y_{12}$ inhibitors	938(92.5)	830(90.9)	0.204	272(90.4)	216(93.1)	0.260
β-Blockers	799(78.8)	689(75.5)	0.082	241(80.1)	182(78.4)	0.647
ACEIs/ARBs	665(65.6)	530(58.1)	<0.001	222(73.8)	160(69.0)	0.224
Statins	997(98.3)	900(98.6)	0.655	294(97.7)	225(97.0)	0.621

Data are presented as mean±SD, median (IQR), n (%), or n/N (%). ACEI, angiotensin-converting enzymes inhibitor; AHI, apnea-hypopnea index; ARB, angiotensin receptor blocker; CABG, coronary artery bypass grafting; DES, drug eluting stent; Hs-CRP, high-sensitivity C-reactive protein; IQR, interquartile range; NSTEMI, non-ST-segment elevation myocardial infarction; ODI, oxygen desaturation index; OSA, obstructive sleep apnea; PCI, percutaneous coronary intervention; SaO_2, arterial oxygen saturation; SD, standard deviation; STEMI, ST-segment-elevation myocardial infarction; TIMI, thrombolysis in myocardial infarction.

Table S3. Crude Number of all Events overall and by number of cardiovascular risk factors.

Variables	All (N=1927)	No SMuRF (N = 130)	1-2 SMuRFs (N = 1264)	3-4 SMuRFs (N = 533)	P value
MACCE	389(20.2)	25(19.2)	256(20.3)	108(20.3)	0.961
Cardiovascular death	33(1.7)	2(1.5)	23(1.8)	8(1.5)	0.882
Myocardial infarction	51(2.6)	2(1.5)	34(2.7)	15(2.8)	0.709
Stroke	43(2.2)	3(2.3)	21(1.7)	19(3.6)	0.044
Ischemia-driven revascularization	159(8.3)	14(10.8)	102(8.1)	43(8.1)	0.558
Hospitalization for unstable angina	272(14.1)	20(15.4)	181(14.3)	71(13.3)	0.781
Hospitalization for heart failure	21(1.1)	1(0.8)	14(1.1)	6(1.1)	0.935
Composite of major cardiovascular events	114(5.9)	5(3.8)	68(5.4)	41(7.7)	0.097
Composite for cardiac events	353(18.3)	22(16.9)	239(18.9)	92(17.3)	0.650
All repeat revascularization	227(11.8)	21(16.2)	140(11.1)	66(12.4)	0.204
All death	46(2.4)	3(2.3)	33(2.6)	10(1.9)	0.647

Composite end point of major cardiovascular events included cardiovascular death, myocardial infarction, and stroke; Composite for cardiac events included cardiovascular death, myocardial infarction, ischemia-driven revascularization, or hospitalization for unstable angina or heart failure. CI, confidence interval; MACCE, major adverse cardiovascular and cerebrovascular event; OSA, obstructive sleep apnea.

Table S4. Crude Number of all Events of OSA versus non-OSA groups overall and in patients with 3-4 SMuRFs.

	All (N=1927)			3-4 SMuRFs (N = 533)		
**Variables**	**OSA (n=1014)**	**Non-OSA (n=913)**	***P* value**	**OSA (n=301)**	**Non-OSA (n=232)**	***P* value**
MACCE	227(22.4)	162(17.7)	0.011	68(22.6)	40(17.2)	0.128
Cardiovascular death	19(1.9)	14(1.5)	0.565	3(1.0)	5(2.2)	0.465
Myocardial infarction	33(3.3)	18(2.0)	0.080	11(3.7)	4(1.7)	0.182
Stroke	25(2.5)	18(2.0)	0.464	12(4.0)	7(3.0)	0.550
Ischemia-driven revascularization	94(9.3)	65(7.1)	0.087	30(10.0)	13(5.6)	0.067
Hospitalization for unstable angina	155(15.3)	117(12.8)	0.120	45(15.0)	26(11.2)	0.207
Hospitalization for heart failure	11(1.1)	10(1.1)	0.982	4(1.3)	2(0.9)	0.926
Composite of major cardiovascular events	72(7.1)	42(4.6)	0.020	25(8.3)	16(6.9)	0.545
Composite for cardiac events	205(20.2)	148(16.2)	0.023	57(18.9)	35(15.1)	0.243
All repeat revascularization	129(12.7)	98(10.7)	0.176	39(13.0)	27(11.6)	0.647
All death	23(2.3)	23(2.5)	0.719	4(1.3)	6(2.6)	0.460

Composite end point of major cardiovascular events included cardiovascular death, myocardial infarction, and stroke; Composite for cardiac events included cardiovascular death, myocardial infarction, ischemia-driven revascularization, or hospitalization for unstable angina or heart failure. CI, confidence interval; MACCE, major adverse cardiovascular and cerebrovascular event; OSA, obstructive sleep apnea.

Figure S1. Percentage of OSA in patients stratified by number of SMuRFs. OSA, obstructive sleep apnea; SMuRF, standard modifiable risk factor.

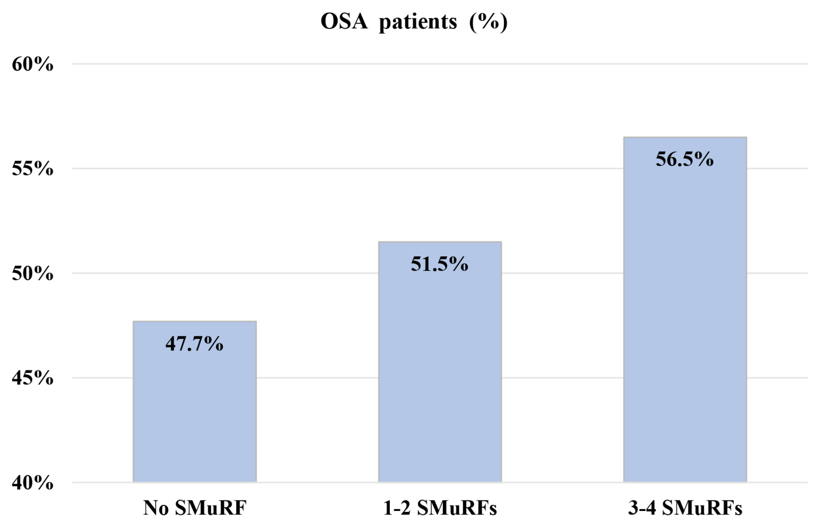

Figure S2. Cumulative Incidence of MACCE by SMuRFs. MACCE, major adverse cardiovascular and cerebrovascular event; SMuRF, standard modifiable risk factor.

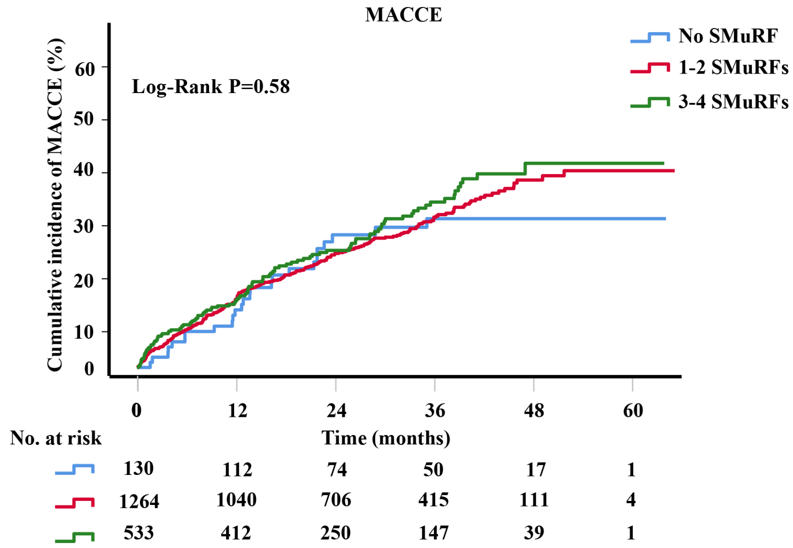

Supplement: Supplementary file 1 — Supplementary Material 1 [file 11239_2023_2830_MOESM1_ESM.pdf]
